# Mito-nuclear discordance within Anthozoa, with notes on unique properties of their mitochondrial genomes

**DOI:** 10.1038/s41598-023-34059-1

**Published:** 2023-05-08

**Authors:** Andrea M. Quattrini, Karen E. Snyder, Risa Purow-Ruderman, Isabela G. L. Seiblitz, Johnson Hoang, Natasha Floerke, Nina I. Ramos, Herman H. Wirshing, Estefanía Rodriguez, Catherine S. McFadden

**Affiliations:** 1 Department of Invertebrate Zoology, National Museum of Natural History, Smithsonian Institution, 10th St. & Constitution Ave. NW, Washington, DC 20560 USA; 2 Department of Biology, Harvey Mudd College, Claremont, CA 91711 USA; 3 Centre for Marine Biology, University of São Paulo, São Sebastião, 11612-109 Brazil; 4 Department of Zoology, Institute of Biosciences, University of São Paulo, São Paulo, 05508-900 Brazil; 5 Division of Invertebrate Zoology, American Museum of Natural History, Central Park West at 79th Street, New York, NY 10024 USA

**Keywords:** Molecular evolution, Phylogenetics, Zoology

## Abstract

Whole mitochondrial genomes are often used in phylogenetic reconstruction. However, discordant patterns in species relationships between mitochondrial and nuclear phylogenies are commonly observed. Within Anthozoa (Phylum Cnidaria), mitochondrial (mt)-nuclear discordance has not yet been examined using a large and comparable dataset. Here, we used data obtained from target-capture enrichment sequencing to assemble and annotate mt genomes and reconstruct phylogenies for comparisons to phylogenies inferred from hundreds of nuclear loci obtained from the same samples. The datasets comprised 108 hexacorals and 94 octocorals representing all orders and > 50% of extant families. Results indicated rampant discordance between datasets at every taxonomic level. This discordance is not attributable to substitution saturation, but rather likely caused by introgressive hybridization and unique properties of mt genomes, including slow rates of evolution driven by strong purifying selection and substitution rate variation. Strong purifying selection across the mt genomes caution their use in analyses that rely on assumptions of neutrality. Furthermore, unique properties of the mt genomes were noted, including genome rearrangements and the presence of *nad5* introns. Specifically, we note the presence of the homing endonuclease in ceriantharians. This large dataset of mitochondrial genomes further demonstrates the utility of off-target reads generated from target-capture data for mt genome assembly and adds to the growing knowledge of anthozoan evolution.

## Introduction

Mitochondrial (mt) genes have a long history of use for phylogenetic reconstruction in animals^1^, and the relative ease with which complete mt genomes can now be obtained has fueled an increase in their use to resolve phylogenetic relationships within many groups^2–4^. Animal mt genomes typically include a highly conserved set of protein-coding genes with few non-coding intergenic regions; are inherited uniparentally without undergoing recombination; and in many cases have rates of substitution that may be an order of magnitude higher than those of the nuclear genome^5^. While these properties might be advantageous for phylogenetic reconstruction in some cases, they may also generate phylogenetic signals that differ from those of the nuclear genome. Discordance between nuclear and mt gene phylogenies is common and can result from biological processes such as introgression or incomplete lineage sorting (ILS) that act differently on mt vs. nuclear genomes (e.g.^6–8^). Alternatively, apparent mt-nuclear discordance can arise from inaccurate estimation of phylogenies due to low statistical power, poor model fit or taxon sampling issues^8^. Recent advances in computational models and increased taxon sampling of both mt and nuclear genomes have allowed these alternative sources of discordance to be evaluated in several well-sampled vertebrate taxa^6,8^. Studies have concluded that mt-nuclear discordance more often arises from biological processes such as introgression and ILS and persists even when factors that lead to inaccurate phylogenetic estimation have been addressed^6–8^.

Phylogenies of anthozoan cnidarians (e.g., corals and sea anemones) reconstructed from mt genes or genomes have often recovered relationships within and among orders that differ from those inferred from both nuclear genes and morphology. The mt genomes of these non-bilaterian metazoans have several unusual properties that are not found in bilaterians^9^. For example, the mt genomes of class Hexacorallia (e.g., sea anemones, scleractinian corals and black corals) encode the standard 13 protein-coding genes found in bilaterians, but only two tRNAs (trnW, trnM)^10–14^. Many hexacorals have group I introns in *nad5* or *cox1*^10–13^, and the latter gene may have a LAGLI-DADG type homing endonuclease encoded within it^13^. The ceriantharian tube anemones have multipartite linear mt genomes^15^. All members of class Octocorallia (e.g., soft corals, gorgonians and sea pens) have just a single tRNA (trnM), but with only one known exception (i.e., a member of genus *Pseudoanthomastus*^16^) their mt genomes include an additional protein-coding gene that encodes the DNA mismatch repair protein, *mtMutS*^17^. At least one sea pen has a bipartite circular mt genome^18^, and other octocoral lineages have undergone frequent rearrangements (inversions) of gene order by a mechanism that appears to involve intramolecular recombination^19–21^.

The unusual property of anthozoan mt genomes that has most impacted their utility for phylogenetic reconstruction is, however, the rate at which they evolve. Unlike bilaterian mt genomes that tend to evolve 5–10X faster than the nuclear genome^22,23^, anthozoan mt genes typically evolve 10–100X slower than nuclear genes^24^. As a result, mt genes that have been widely used in bilaterians for barcoding, species-level phylogenetic analyses and phylogeography are often invariant within—and sometimes between—anthozoan genera^25,26^. These slow rates of mt gene evolution have, however, increased the potential utility of mt genes for reconstructing deep phylogenetic relationships among the families and orders of Anthozoa, a group of organisms that last shared a common ancestor in the pre-Cambrian^27,28^. Nonetheless, phylogenies of Anthozoa reconstructed from complete mt genomes (or their protein-coding genes) have often been incongruent with other sources of morphological and phylogenomic evidence. The most notable of these discrepancies has been a lack of support for the monophyly of the anthozoan classes, Hexacorallia and Octocorallia. Mitochondrial phylogenies have often placed Octocorallia sister to the cnidarian sub-phylum Medusozoa^4,21,29,30^, despite the very strong morphological and life-history evidence for the monophyly of Anthozoa (see^31^), which has also been confirmed in several phylogenomic studies^32,33^. Moreover, in some of these same analyses Hexacorallia has been recovered outside of Cnidaria, as the sister to a clade of sponges^4,34^. Mitochondrial gene phylogenies have also recovered Ceriantharia (tube anemones) sister to the rest of Anthozoa^15,30,35^ rather than within Hexacorallia as supported by genomic-scale studies^27,28,32^. In addition, previous studies have suggested that Scleractinia is paraphyletic with Corallimorpharia^4,12,36^) and have differed from nuclear gene phylogenies in the placement of the orders Actiniaria, Zoantharia and Antipatharia and in the relationships among the major clades of Scleractinia^37,38^. Within Octocorallia, mt genes and/or genomes have provided little statistical support for the deepest nodes in either of the two major clades that have been recognized^29,30,39,40^.

Explanations that have been proposed to explain the incongruence between mt and nuclear or morphological phylogenies of Anthozoa include substitution saturation of the mt genome^21,36,41^, rate heterogeneity between the major lineages^29^, and long branch attraction (LBA) due to the combined effects of rate heterogeneity and incomplete or biased taxon sampling^34^. Most mt genome phylogenies and phylogenomic analyses of anthozoans published to date have been taxon-sparse, often omitting entire orders^29,32,33^ or have drawn comparisons between topologies generated from completely different taxon sets^41^. As a result, it is still unclear if the source of incongruence between mt and nuclear gene phylogenies of anthozoans is simply an artifact of incomplete, biased and incomparable taxon sampling or if the evolutionary signal present in anthozoan mt genomes does indeed differ from that of the nuclear genome.

Recent advances in phylogenomic methods and technologies have facilitated the ability to obtain complete mt genomes while simultaneously generating sequence reads for thousands of nuclear genes. In particular, target-enrichment methods used to sequence ultraconserved elements (UCEs) and exonic regions of the nuclear genome can recover complete or near-complete mt genomes as off-target reads^3^. Comparisons of mt vs nuclear gene phylogenies from the same set of taxa (often the same individuals) facilitate investigation of the causes of mt-nuclear incongruence by eliminating artifacts that may be caused by unequal or different taxon sampling.

In recent phylogenomic analyses of Anthozoa based on UCEs and exons^27,28^, complete or near-complete mt genomes were recovered for a majority of the taxa sequenced. Here, we used the complete set of mt protein-coding sequences to reconstruct the phylogenies of the Octocorallia and Hexacorallia classes and compared those to nuclear gene phylogenies generated for the same set of individuals. The dataset comprised a total of 202 species representing all orders and > 50% of extant families. With this comparable dataset, the impacts of sampling biases were removed and we were able to robustly explore whether incongruence is related to evolutionary signal. New findings on the unique properties of the recovered mt genomes are also noted.

## Methods

### Target-enrichment analyses

UCE and exon loci were target enriched and bioinformatically extracted from high-throughput sequencing data as described in Quattrini et al.^27,42^ using the anthozoa-v1 baitset^42^. Briefly, raw reads were cleaned using illumiprocessor^43^ and Trimmomatic v 0.35^44^ and then assembled using either Spades v 3.1 (^45^; with the –careful and –cov-cutoff 2 parameters) or Trinity v. 2.0^46^. The phyluce pipeline was then used as described in the online tutorials (https://phyluce.readthedocs.io/en/latest/tutorials/tutorial-1.html) with some modifications (see supplemental code in 27, 42). Using phyluce, 75% and 50% taxon-occupancy matrices were created for each nuclear locus, aligned with MAFFT v7.130b^47^, and loci were concatenated (*phyluce_align_format_nexus_files_for_raxml*) separately for hexacorals (n = 108) and octocorals (n = 94).

### Mt genome analyses

Whole and partial mt genomes were extracted from the off-target reads in the target-enrichment sequencing data. Mitochondrial genomes were extracted and assembled in three ways. First, we used blastn to find whole or partial genomes in the Trinity or Spades assemblies and then extracted those as fasta sequences. Second, we used Novoplasty v 2.6^48^ to assemble mt genomes using the adapter-trimmed paired-end reads. Seed files were used to help assemble each species and consisted of *cox1* sequences downloaded from GenBank for the species of interest or a closely-related species. Third, Geneious Prime 2020 (https://www.geneious.com) was used for genomes that were difficult to assemble with Spades and Novoplasty. The *Map to Reference* tool, with Mapper set to “Geneious” and Sensitivity to “Medium Sensitivity/Fast”, within Geneious was used. Reference sequences included individual mt loci from closely-related taxa, either *mtMutS, cox1* or *16S*. The fine-tuning option required iterations from “up to 5” to “up to 25” times to assemble the complete mt genome from the reference sequences.

Following mt genome assembly, fasta files were uploaded to Mitos2 (^49^, http://mitos2.bioinf.uni-leipzig.de) for annotation (translation code = 4). For further analyses, we used only species whose mt genomes were represented by at least 50% of the protein coding genes (hexacorals n = 108, octocorals n = 94, Suppl. Table 1), except that we included five ceriantharians with low mt genome recovery (e.g., for 15–53% of genes recovered for each species). Protein-coding genes were then each aligned separately using MAFFT v7.130b^47^ and adjusted by eye to ensure the sequences were in frame. Loci were then concatenated with *phyluce_align_concatenate_alignments.*

Some mt genomes for which we had corresponding nuclear data could not be assembled, or were published in previous studies, and so sequences were downloaded from GenBank and subsequently used in our analyses (Suppl. Table 1). We used mt data from GenBank for 26 hexacorals; 16 of these were of the same individuals used in our study. All octocoral mt genomes were also assembled concurrently in another study^16^ and added to GenBank by those authors.

### Phylogenomic analyses

Removing loci that are saturated can improve phylogenomic analyses^50^. Therefore, we ran saturation tests on each of the different locus datasets using Phylomad^51^. For nuclear loci, we ran saturation tests using models of entropy on all variable sites and only on those that had no missing data in each locus alignment following^50,51^. Entropy values below the predicted threshold value indicate a high risk of substitution saturation. Datasets are denoted hereafter as LR (low risk loci) and LRM (low risk loci with no missing data in saturation test). For the mt data, we ran saturation tests on sites with no missing data for the concatenated alignment. Loci with substitution saturation were removed and then various datasets were used for further phylogenetic analyses (Suppl. Table 2, Table 1).

**Table 1:** Summary statistics for different alignment datasets.

Dataset	Taxon Occupancy	# Loci	Alignment Size	# BS > 95	# BS 95–80	# BS < 80	# SH > 95	# SH 95–80	# SH < 80
Hexacorallia									
ASTRAL	75%	222	NA	95*	2*	9*	NA	NA	NA
ASTRAL LR	75%	149	NA	89*	4*	13*	NA	NA	NA
ASTRAL LRM	75%	95	NA	92*	4*	10*	NA	NA	NA
75	75%	222	85,327	100	3	3	101	1	5
75LR	75%	149	66,567	100	3	3	100	1	4
75LRM	75%	95	38,534	93	7	6	96	2	8
50	50%	756	246,027	103	2	1	103	2	1
50LR	50%	545	196,468	103	1	2	103	1	2
50LRM	50%	380	126,183	99	6	1	100	3	3
Mt	94–98%	13	12,465	94	7	5	83	11	12
Octocorallia									
ASTRAL	75%	621	NA	83*	4*	4*	NA	NA	NA
ASTRAL LR	75%	575	NA	83*	1*	7*	NA	NA	NA
ASTRAL LRM	75%	408	NA	76*	7*	8*	NA	NA	NA
75	75%	621	305,233	82	3	6	88	2	1
75LR	75%	575	290,130	85	2	4	87	2	2
75LRM	75%	408	213,477	80	8	4	85	4	2
50	50%	1252	555,701	82	5	4	87	2	2
50LR	50%	1161	529,092	85	2	4	85	1	5
50LRM	50%	827	385,627	85	4	2	86	3	2
Mt	77–100%	14	16,176	69	14	8	69	12	10

*BS* bootstrap, *SH* Shimodaira-Hasegawa approximate likelihood ratio test, *LRM* low risk loci only with no missing data in saturation test, *LR* low risk loci only while allowing for missing data in saturation test, *mt* mitochondrial alignment.

*Posterior probability.

Selection tests were conducted using codon-based models in Codeml within PAML v. 4^52^. The one ratio model (M0) was run on the mt alignment only, for both octocorals and hexacorals. This allowed us to estimate average omega (*dN/dS*) and kappa (*ts/tv*) values across all branches in the corresponding mt phylogenies. Omega values = 1 indicate the locus is evolving neutrally, values > 1 indicate positive selection and values < 1 indicate negative or purifying selection. Higher kappa values indicate transition relative to transversion bias.

Phylogenomic analyses were conducted using maximum likelihood in IQTree v 2.1^53^ on each of the concatenated datasets (Table 1). We ran partitioned analyses on the different datasets using the best model for each locus (-m TESTMERGE) chosen with ModelFinder^54^. Ultrafast bootstrapping (-bb 1000^55^) and the Sh-like approximate likelihood ratio test (-alrt 1000^56^) were conducted as well as site-concordance factors (-scfl 100)^57^. For nuclear data, a species tree analysis was also conducted using ASTRAL III v 5.7, which is statistically consistent under a multispecies coalescent model^58^. A tree for each locus was constructed in IQTree using the best fit model of evolution selected with ModelFinder for each locus. We used the 75% taxon-occupancy data matrices for octocorals and for hexacorals. Treeshrink^59^ was used to remove long branches, and the newick utility, nw_ed, was used to remove branches with < 30% bootstrap support prior to running ASTRAL. Site concordance factors (-scfl 100) were also calculated on the ASTRAL species tree but using the concatenated alignment of the loci used in the species tree analysis. The phylogenetic relationship of *Renilla muelleri* to other octocorals was spuriously placed in some phylogenies. Because this species is well-supported in Pennatuloidea, we pruned this species from all phylogenetic trees using the R *phytools* package^60^.

Following phylogenetic inference, we conducted Robinson-Foulds distance (R-F,^61^) tests using IQTree v2.1 (-rf). R-F distances were calculated between all pairs of hexacoral unrooted trees and all pairs of octocoral unrooted trees. We also used the R *TreeDist*^62^ package to calculate generalized Robinson-Foulds (gR-F) distances as a comparison. The two most congruent mt and nuclear trees based on maximum likelihood were determined from the smallest R-F distances for both hexacorals and octocorals and plotted. In cases where R-F distances were the same, we chose the topology with lower gR-F distances that also had the most nodes with bs support values > 95%. Hexacorals were rooted at the Ceriantharia based on prior phylogenomic studies of the phylum Cnidaria^32,33^ and Scleralcyonacea was rooted to Malacalcyonacea based on prior phylogenomic studies^27,28,40^.

GC content (%) was calculated for all mt and nuclear loci to determine if GC content contributed to mt-nuclear discordance. The program SeqKit^63^ was used to calculate the GC content at each locus for every individual. GC content was then averaged over all loci for each individual using *awk* and plotted in R using *ggplot2*^64^*.* A one-way analysis of variance was conducted in R to test whether GC content differed significantly between mt and nuclear data. Code for all analyses can be found in Suppl. File S1 and all trees and alignments can be found on figshare.

## Results

### Mt genome assemblies

Herein we assembled complete or near complete mt genomes of 75 hexacorals (73X average coverage) from the following orders: Actiniaria, Antipatharia, Ceriantharia, Corallimorpharia, and Scleractinia. Ceriantharian mt genomes were difficult to assemble*.* Out of five ceriantharians, none had complete mt genomes and only two genes were found for one species (*Ceriantheomorphe brasiliensis*)*.* Only one species, *Botruanthus mexicanus,* had a near complete genome assembly. We confirmed the presence of group I introns (Suppl. Table 3) in many taxa. In Actiniaria, Antipatharia, Zoantharia, and *Relicanthus daphneae,* two protein-coding genes, *nad1* and *nad3*, were found inserted as introns within *nad5.* Ten protein-coding genes were found in the *nad5* intron of most scleractinians, with the exception of *Caryophyllia arnoldi* in which we found only seven protein-coding genes and *rns* within the *nad5* intron. In Corallimorpharia, 10 protein-coding genes were in the *nad5* intron of *Corallimorphus profundus,* and for the rest of the corallimorpharians (*Rhodactis osculifera, Discosoma carlgreni,* and *Ricordea florida)*, all genes but *trnW* were in the *nad5* intron*.* Another group 1 intron that encodes a homing endonuclease from the LAGLI-DADG family was present in *cox1* of some hexacorals (uploaded to figshare). We confirmed the presence of this endonuclease in 24% of actiniarians, 28% of scleractinians, 17% of antipatharians, and 100% of corallimorpharians (Suppl. Table 3). We also documented this intron in two species of Ceriantharia, *Botruanthus mexicanus* and *Ceriantheomorphe brasiliensis*.

Of the complete (or near complete) mt genomes of hexacorals assembled in this study, only three species displayed gene order rearrangements relative to other taxa in their respective orders (Suppl. Table 3). Within Actiniaria, only one species sequenced, *Alicia sansibarensis,* exhibited a mt genome rearrangement with *cox2-nad4-nad6-cob* inserted prior to *atp8* instead of between *nad6* and *rns.* Of the scleractinians, *Caryophyllia arnoldi* had a genome rearrangement with the *cob-nad2-nad6* gene block inserted after the 3’ end of *nad5* instead of within the *nad5* intron*.* The mt genome of *Madrepora oculata* also had a gene rearrangement, with a switch in the order of *cox2* and *cox3* compared to all other scleractinians. *Corallimorphus profundus* also had a different genome rearrangement compared to *R. osculifera*, *D. carlgreni,* and *R. florida* (Suppl. Fig. 3). *Corallimorphus profundus* had 10 protein-coding genes and *rns* within the *nad5* intron. In contrast, *R. osculifera, D. carlgreni* and *R. florida* have all other genes but *trnW* within the *nad5* intron.

### Alignment summary

For hexacorals, concatenated nuclear locus alignments across 50–75% taxon-occupancy datasets ranged from 38,534 to 246,027 bp with 95 to 756 loci in each dataset (Table 1). For each hexacoral species, locus recovery (average read coverage = 15X) ranged from 303 to 1156, with overall few loci (342 to 589) recovered in ceriantharians (Suppl. Table 1). For octocorals, concatenated nuclear locus alignments across 50–75% taxon-occupancy datasets ranged from 213,477 to 555,701 bp with 408 to 1,252 loci (Table 1). For each octocoral species, 604 to 1275 loci were recovered (average read coverage = 26X) (Suppl. Table 1).

All 13 protein-coding genes were included in the alignment for 79% of all hexacoral species (Suppl. Tables 1 and 3). The hexacoral mt genome alignment containing the 13 protein-coding genes was 12,465 bp, and for each gene at least 94–98% of the species were represented.

For octocorals, all 14 protein-coding genes were included in the alignment for 80% of species. The octocoral mt genome alignment was 16,176 bp, and for each gene 96–100% of the species were represented, except for *mtMutS*. *mtMutS* was included for only 77% of the species as for some species *mtMutS* was highly incomplete or, in some cases (< 5%), it could not be reliably aligned to other species.

**Figure 1:**
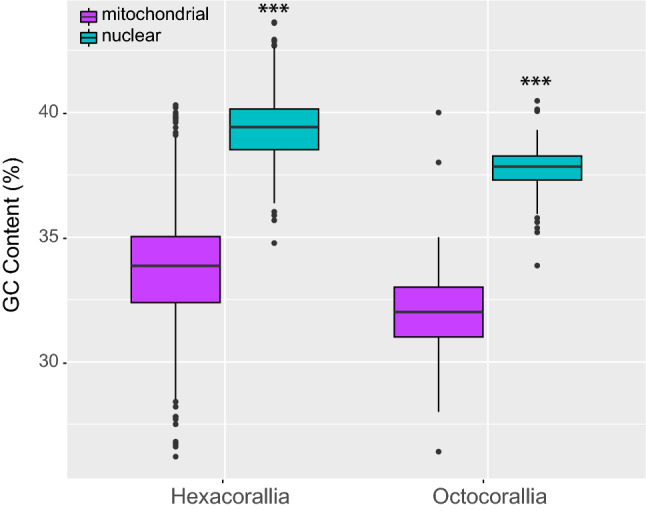
GC content (%) in nuclear and mitochondrial loci of hexacorals and octocorals. ***p < 0.001.

**Figure 2:**
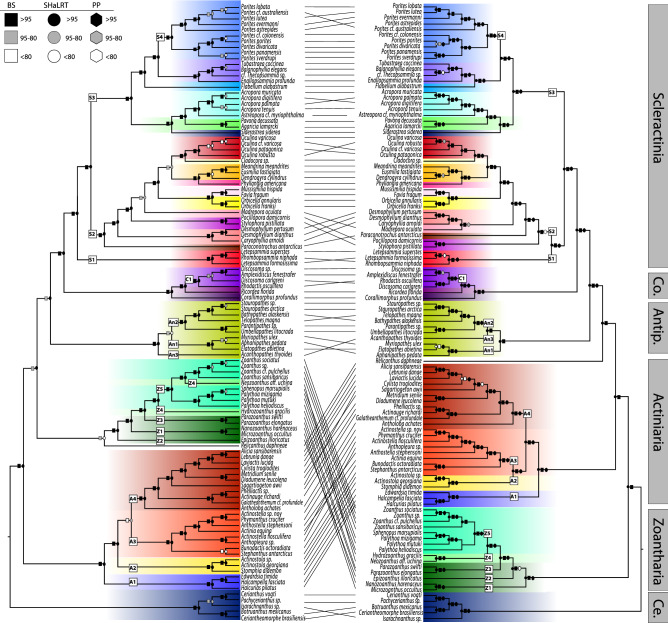
Maximum likelihood tree of Hexacorallia inferred from (left) mitochondrial and (right) nuclear loci (50% taxon occupancy, no loci with substitution saturation as denoted in saturation tests with no missing data included). *BS* Ultrafast bootstraps, *SHaLRT* Shimodaira-Hasegawa approximate likelihood ratio test, *PP* posterior probabilities from the most similar ASTRAL tree. Numbered squares on branches identify clades discussed in “Results”.

GC content differed between mt and nuclear loci for both hexacorals and octocorals (Fig. 1). GC content was significantly higher (F = 246, p < 0.001) across nuclear loci (39%) than mt loci (34%) in hexacorals. Of note, the GC content in the mt genes of Zoantharia was more similar to the GC content of the nuclear loci, and slightly higher by 0.5%. This pattern contrasted with the other hexacoral orders in which mt GC content was 4–8% lower than nuclear GC content. Ceriantharia had low GC content in mt data as compared to other orders. Antipatharia had slightly higher GC content (42%) in the nuclear loci compared to other groups of hexacorals (38–40%). In octocorals, GC content was also significantly higher (F = 779, p < 0.001) in nuclear loci (38%) as compared to mt loci (32%). However, two octocoral species had mt GC content similar to the nuclear data, at 38–40%: *Leptophyton benayahui* and *Tenerodus fallax,* which are sister to one another in both phylogenies and on long branches in the mt tree (Fig. 2).

Selection tests on the mt genome alignments indicated that the mt genomes are under strong purifying selection. The omega value (*dN/dS*) for hexacorals was 0.10 while the value for octocorals was 0.14. The kappa value (*ts/tv*) for hexacorals was 2.7, whereas in octocorals it was higher at 3.9; both values indicate higher numbers of transitions than transversions. Saturation tests conducted using PhyloMad indicated that neither the hexacoral nor octocoral mt alignment was under saturation as indicated by entropy tests (Suppl. Fig. 1, Suppl. Table 2). For nuclear-locus datasets, 8–50% of the loci in each dataset had a high risk of substitution saturation. Hexacorals tended to have more saturated loci, with 30–50% saturated loci per dataset whereas octocorals had a lower number, with 8–35% saturated loci per dataset.

### Mt-nuclear discordance

#### Hexacorallia

Overall, all phylogenies constructed for Hexacorallia were well supported (Table 1). Among all nuclear trees constructed with ASTRAL and IQTree, 83 to 97% of nodes (106 total nodes) on each tree had higher than 95% ultrafast bootstrap (bs) values, posterior probabilities (pp), and SH-aLRT values. Similarly, the mt genome tree was well supported with 78–89% of nodes having higher than 95% ultrafast bs values and SH-aLRT values.

There were some differences among all hexacoral phylogenies, but nuclear phylogenies constructed with ASTRAL and IQTree were mostly congruent with one another (pairwise R-F = 4–28, gR-F = 0.01–0.08). The R-F distances between the hexacoral mt genome tree and the nuclear trees, however, were much larger, ranging from 56 to 68 (gR-F = 0.14–0.16). The mt genome tree was most similar to the ASTRAL species trees (R-F = 56–60, gR-F = 0.14–0.15) compared to the maximum likelihood phylogenies (R-F = 62–66, gR-F = 0.14–0.16), although the differences were negligible. There were three maximum likelihood phylogenies that were all equally congruent with the mt genome phylogeny (R-F = 62, gR-F = 0.15–0.16). Of the three most congruent ML trees, the tree with 50% data occupancy and highly saturated loci removed without missing data in the saturation tests (50LRM) had the lowest gR-F value and highest BS support values.

There were a few species on long branches in the mt genome tree, but not the nuclear tree, including the zoantharians *Nanozoanthus harenaceus* and *Microzoanthus occultus* and the scleractinian *Paraconotrochus antarcticus* (see Suppl. Files, Suppl. Fig. 2). In addition, the branch lengths at the tips were much shorter in the mt genome tree compared with the nuclear tree, indicating slow substitution rates between conspecifics and in many cases, between genera. Overall, however, the rate variation appeared higher in the mt genome tree as compared to the nuclear tree (Suppl. Fig. 2).

Although there were several differences among shallow nodes in all topologies, two major differences were apparent at deep nodes (Fig. 2). First, the relationship of Zoantharia and Actiniaria to other orders differed among topologies. In the mt genome tree, Actiniaria was sister to all other hexacoral orders except Ceriantharia (bs = 100, SHaLRT = 100, sCF = 82). This same relationship was also recovered in the ASTRAL species trees (bs = 100, SHaLRT = 100, sCF = 54–56) and the 75LRM tree (bs = 100, SHaLRT = 100, sCF = 56, Fig. 2, see Suppl. Files). In contrast, in most of the maximum likelihood trees for the nuclear dataset, Zoantharia diverged earlier than Actiniaria (bs = 100, SHaLRT = 100, pp = 100, sCF = 52–84). Second, the relationship of *Relicanthus daphneae* to other orders differed among phylogenies. In the mt genome phylogeny, *R. daphneae* was sister to the zoantharians, although with low to moderate support (UF = 88, SHaLRT = 77, sCF = 42). In the majority of nuclear phylogenies, *R. daphneae* was also recovered as sister to Antipatharia-Corallimorpharia-Scleractinia, with variable support depending on dataset (bs > 84, SHaLRT > 75, pp > 41, sCF > 32).

There were also some differences between mt genome and nuclear phylogenies within each hexacoral order. Within Scleractinia, there were differences among trees at the shallow nodes, including branch lengths and the relationships among species of *Porites* (S4 clade)*,* but the major difference was the placement of the family Micrabaciidae (S1). Nuclear phylogenies all strongly supported that this family is sister to the Robust/Vacatina clade (S2) of Scleractinia (bs = 100, SHaLRT = 100, pp = 97–100, sCF = 37–40). In contrast, Micrabaciidae (S1) was recovered as sister to all other Scleractinia (S2 + S3) with strong support (bs = 100, SHaLRT = 99, sCF = 33) in the mt genome phylogeny. Within Actiniaria, the position of the superfamily Actinostoloidea (A2) differed between mt genome and nuclear phylogenies. This superfamily was sister to the superfamilies Metridioidea + Actinioidea (A4 + A3) in the mt genome phylogeny (bs = 100, SHaLRT = 100, sCF = 48.6) whereas it was sister to the superfamily Actinioidea (A3) in all nuclear phylogenies (bs = 100, SHaLRT = 99–100, pp = 100, sCF = 37–38). Within Antipatharia, the position of *Acanthopathes thyoides* (An3) differed between mt genome and nuclear phylogenies. This species was sister to all other antipatharians (An1 + An2) in the mt genome phylogeny (bs = 100, SHaLRT = 100, sCF = 72) whereas it was sister to the family Schizopathidae (An2) in the majority of nuclear phylogenies (bs = 76–100, SHaLRT = 23–100, pp = 91–98, sCF = 34–37), except for the 50LRM and ASTRAL LRM topologies (bs = 100, SHaLRT = 100, pp = 100, sCF = 65–67), which matched the mt genome tree. Within Zoantharia, the placement of *Epizoanthus illoricatus* (Z2) and *Neozoanthus* aff*. uchina* (Z4 in part) differed among mt genome and nuclear phylogenies. In the mt genome tree, *E. illoricatus* (Z2) was sister to the rest of the zoantharians (bs = 100, SHaLRT = 100, sCF = 66), whereas in all nuclear phylogenies, *Nanozoanthus harenaceus* and *Microzoanthus occultus* (Z1) were sister to the rest of the zoantharians (bs = 100, SHaLRT = 100, pp = 100, sCF = 37–38). *Neozoanthus.* aff. *uchina* (Z4 in part) was sister to the family Zoanthidae (Z5) in the mt genome phylogeny (bs = 100 SHaLRT = 100, sCF = 49) whereas it was sister to *Hydrozoanthus gracilis* (Z4) in all nuclear phylogenies (bs = 100 SHaLRT = 100, pp = 100, sCF = 48–51). Within Corallimorpharia, there were differences within the Discosomidae family (C1) with *Rhodactis osculifera* sister to *Discosoma carlgreni* in the nuclear phylogeny yet sister to the remaining discosomids in the mt genome phylogeny.

#### Octocorallia

Nuclear gene phylogenies for Octocorallia were in general well supported. Among all nuclear trees constructed with ASTRAL and IQTree, 83 to 96% of nodes (91 total nodes) on each tree had higher than 95% ultrafast bootstrap (bs) values, posterior probabilities (pp), and SH-aLRT values. In contrast, mt genome trees for Octocorallia were not as well supported with only 76% of nodes having higher than 95% ultrafast bs and SHaLRT values.

Nuclear phylogenies constructed with ASTRAL and IQTree were somewhat congruent with one another (R-F = 6–36, gR-F = 0.03–0.16 Table 2, see Suppl. Files). The R-F distances between the octocoral mt genome tree and the nuclear trees, however, were much larger, ranging from 60 to 72 (gR-F = 0.23–0.36). Octocoral mt genome trees were somewhat more similar to the maximum likelihood phylogenies (R-F = 60–68, gR-F = 0.23–0.28) as compared to the ASTRAL trees (R-F = 68–72, gR-F = 0.25–0.36). The most similar tree (R-F = 60, gR = F = 0.23) to the mt genome phylogeny was constructed with a 75% taxon occupancy data matrix with highly saturated loci removed and no missing data in the saturation test (75LRM). In general, branch lengths differed between mt genome and nuclear trees (Suppl. Fig. 2) and rate variation was higher across the mt genome tree. The branch lengths at the tips were much shorter in the mt genome tree compared with the nuclear tree, but there were also several long branches recovered in the mt genome tree. In the mt genome tree, seven species were on very long branches (*Muricella* sp., *Leptophyton benayahui, Tenerodus fallax, Cornularia pabloi, Pseudoanthomastus* sp., *Erythropodium caribaeorum*, and *Melithaea erythraea*), a pattern not recovered in nuclear phylogenies (see Suppl. Files, Suppl. Fig. 2).

**Table 2:** Pairwise Robinson-Foulds and Generalized Robinson-Foulds (in parentheses) distances between hexacoral topologies and octocoral topologies.

	ASTRAL	ASTRAL LR	ASTRAL LRM	75	75LR	75LRM	50	50LR	50LRM
Hexacorallia									
ASTRAL	0								
ASTRAL LR	4 (0.01)	0							
ASTRAL LRM	12 (0.04)	12 (0.04)	0						
75	18 (0.05)	20 (0.05)	28 (0.08)	0					
75LR	16 (0.05)	16 (0.05)	26 (0.08)	6 (0.01)	0				
75LRM	26 (0.06)	24 (0.06)	26 (0.07)	20 (0.05)	20 (0.05)	0			
50	14 (0.05)	14 (0.04)	20 (0.07)	14 (0.01)	14 (0.02)	22 (0.05)	0		
50LR	16 (0.05)	14 (0.04)	24 (0.07)	8 (0.01)	4 (0.01)	18 (0.05)	12 (0.02)	0	
50LRM	26 (0.07)	24 (0.06)	24 (0.07)	18 (0.03)	18 (0.03)	22 (0.05)	12 (0.02)	14 (0.03)	0
Mt	58 (0.14)	60 (0.15)	**56 (0.14)**	62 (0.16)	62 (0.15)	66 (0.14)	64 (0.16)	64 (0.16)	**62 (0.15)**
Octocorallia									
ASTRAL	0								
ASTRAL LR	6 (0.03)	0							
ASTRAL LRM	14 (0.06)	12 (0.04)	0						
75	28 (0.14)	32 (0.15)	30 (0.14)	0					
75LR	34 (0.15)	36 (0.16)	36 (0.15)	6 (0.01)	0				
75LRM	30 (0.12)	30 (0.12)	28 (0.12)	14 (0.10)	20 (0.10)	0			
50	24 (0.13)	28 (0.15)	30 (0.14)	10 (0.05)	16 (0.06)	24 (0.13)	0		
50LR	28 (0.14)	32 (0.16)	32 (0.15)	4 (0.03)	10 (0.04)	18 (0.11)	6 (0.03)	0	
50LRM	28 (0.13)	32 (0.14)	32 (0.13)	4 (0.03)	10 (0.04)	18 (0.11)	10 (0.04)	8 (0.05)	0
Mt	72 (0.27)	70 (0.36)	**68 (0.25)**	68 (0.28)	66 (0.27)	**60 (0.23)**	68 (0.28)	66 (0.28)	68 (0.27)

**Figure 3:**
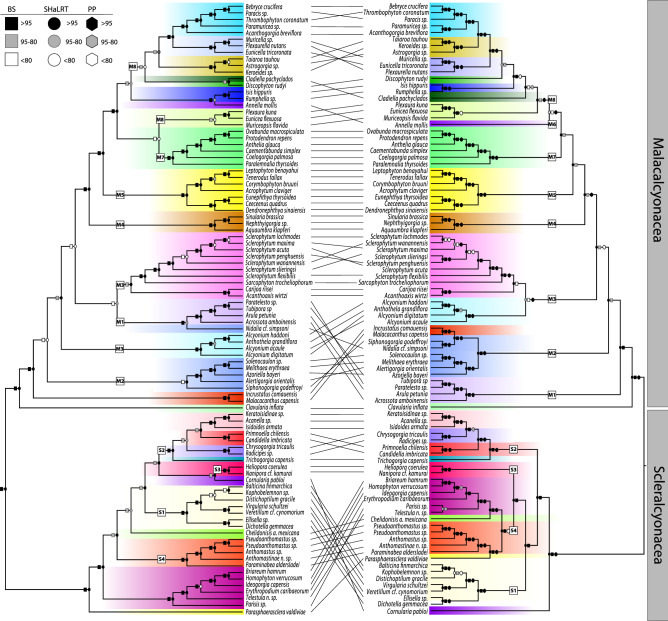
Maximum likelihood tree of Octocorallia inferred from (left) mitochondrial and (right) nuclear loci (75% taxon occupancy, no loci with substitution saturation as denoted in saturation tests without missing data). *BS* Ultrafast bootstraps, *SHaLRT* Shimodaira-Hasegawa approximate likelihood ratio test, *PP* posterior probabilities from the most similar ASTRAL tree. Numbered squares on branches identify clades discussed in “Results”.

Numbers in bold indicate the mt-nuclear comparison used for Figs. 2 and 3.

*LRM* low risk loci only with no missing data in saturation test, *LR* low risk loci only with missing data in saturation test, *mt* mitochondrial alignment, 75 = 75% taxon occupancy dataset, 50 = 50% taxon occupancy dataset.

Numerous differences were apparent among the octocoral mt genome and nuclear phylogenies (Fig. 3). Within the order Scleralcyonacea, the placement of Pennatuloidea + Ellisellidae (clade S1) differed. In the mt genome tree this clade was sister to the Keratoisididae + Primnoidae + Chrysogorgiidae (S2) and Helioporidae (S3) clades (bs = 90, SHaLRT = 100, sCF = 29). In the nuclear datasets, it was sister either to clades S3 + S4 with various levels of support (bs = 53–100, SHaLRT = 25–99, sCF = 33–36) or sister to clade S2 + S3 + S4 with strong support (bs = 100, SHaLRT = 100, pp = 93–100, sCF = 36*). Cornularia pabloi* also changed positions, diverging later (sister to clade S3) in the mt genome phylogeny (bs = 90, SHaLRT = 95, sCF = 28) as compared to all nuclear phylogenies where it was placed sister to all other scleralcyonaceans (bs = 100, SHaLRT = 100, pp = 100, sCF = 37). *Parasphaerasclera valdiviae* was an early-diverging lineage and sister to all other scleralcyonaceans in the mt genome phylogeny (bs = 100, SHaLRT = 100, sCF = 63) whereas it was sister to family Coralliidae in the nuclear phylogeny (bs = 100, SHaLRT = 100, pp = 100, sCF = 37). Helioporidae (S3) was recovered as sister to clade S4 in the maximum likelihood nuclear phylogenies (bs = 99–100, SHaLRT = 99, sCF = 36) but sister to clade S2 in the mt genome phylogeny (bs = 90, SHaLRT = 97, sCF = 34) and the ASTRAL phylogenies, although the relationships in the species trees were poorly to moderately supported (pp = 5–86, sCF = 34). Family Keratoisididae was recovered as sister to Primnoidae in the mt genome phylogeny (bs = 94, SHaLRT = 96, sCF = 32) and in one nuclear phylogeny (50LRM) but with poor support (bs = 79, SHaLRT = 47, sCF = 32). In all other nuclear phylogenies, Keratoisididae was recovered sister to Chrysogorgiidae (bs = 100, SHaLRT = 100, pp = 90–100, sCF = 36–37).

Within Malacalcyonacea, several differences among phylogenetic relationships were noted, including some relationships among congeneric species. The Incrustatidae + Malacacanthidae clade was an early-diverging lineage and sister to most malacalcyonacean families (except for *Clavularia inflata)* in the mt genome tree (bs = 100, SHaLRT = 100, sCF = 50), but these families diverged later as part of the M2 clade in the nuclear phylogenies. The Tubiporidae + Arulidae clade (M1) was sister to all malacalcyonaceans (except for *C. inflata)* in the nuclear phylogeny (75LRM). In the mt genome phylogeny, it included *Nidalia* and was sister to the Sarcophytidae + Carijoidae clade (M3a) (bs = 72, SHaLRT = 86, sCF = 32). An Anthogorgiidae + Eunicellidae + Plexaurellidae clade (M8a) was sister to Paramuriceidae (M8c) in the mt genome phylogeny (bs = 99, SHaLRT = 95, sCF = 35). In contrast, the Keroeididae + Taiaroidae + Astrogorgiidae clade (M8b) was sister to Paramuriceidae (M8c) in the nuclear phylogenies (bs = 99–100, SHaLRT = 99–100, pp = 100, sCF = 34–42). Within Sarcophytidae, relationships differed among species between mt genome and nuclear phylogenies.

## Discussion

### Mt genome properties

Utilizing a total of 202 complete or near-complete mitochondrial (mt) genomes, we were able to examine mt-nuclear discordance within the Anthozoa and explore the unique mt genome properties of all orders belonging to this sub-phylum of Cnidaria. In addition to the mt genomes newly assembled here, most of the previously published mt genomes^16,38,65^ that we included in our analyses had been assembled from the raw sequence data from Quattrini et al.^27,42^. This large dataset of mt genomes further demonstrates the utility of off-target reads generated from target-capture data for the assembly of mt genomes and adds to the growing knowledge of mt genome evolution within the sub-phylum Anthozoa.

Although group I introns have been previously recorded in hexacorals^10–14,65–68^, we note their pervasiveness across the group. A *nad5* intron of at least two protein-coding genes and up to all 13 is present in the majority of hexacoral families. From our data, it also appears that this intron is present in Ceriantharia, however, this needs further confirmation as we had difficulties assembling mt genomes in that order. The other group I intron that encodes a homing endonuclease from the LAGLI-DADG family is present in *cox1* in many hexacorals. Both gains and/or losses of this gene have been previously noted in the hexacoral orders Scleractinia^13^, Corallimorpharia^67^, Actiniaria^68^, and Zoantharia^65^. This endonuclease appears to be more common in some orders (Zoantharia, Corallimorpharia) than others (Scleractinia). Based on annotation from Mitos2, we also documented this intron in two ceriantharians. To our knowledge, this intron has not yet been documented in the order Ceriantharia. Based on its distribution across the phylogeny, the homing endonuclease, likely a result of horizontal transmission^13^, has been gained and lost within Hexacorallia for several hundred million years, with origins dating to 300–400 MYA^27^. To date, no introns have been recorded in Octocorallia.

Mitochondrial genome rearrangements within Anthozoa have been a topic of interest for over two decades, as species in this sub-phylum exhibit several gene order changes. Of the 108 complete (or near complete) mt genomes of hexacorals examined in this study, only 6% displayed gene order rearrangements relative to the canonical gene order within their respective taxonomic order; many of these rearrangements have been described in prior studies (e.g.^12,68,69^). In contrast to hexacorals, octocorals have undergone gene rearrangements more frequently across their phylogenetic history^18–21,70^. Of the 92 complete to near complete octocoral mt genomes used in this study, 21% had gene rearrangements. Brockman and McFadden^20^ suggested that octocoral gene rearrangements evolve via inversions of conserved gene blocks (or intramolecular recombination) whereas hexacoral gene rearrangements are likely caused by gene shuffling. Additionally, they hypothesized that the presence of the mt mis-match repair protein, *mtMutS* (unique to Octocorallia) might play a role in mediating these gene inversions. A recent review by Johansen and Emblem^71^ suggested that the large *nad5* intron that is ubiquitous in hexacorals (but absent from octocorals) perhaps stabilizes mt genome organization in that class. With the increasing availability and decreasing costs of high-throughput sequencing combined with new analytical methods for assembling and annotating mt genomes (e.g., MitoFinder,^3^), many new discoveries likely await regarding the mt genome evolution of anthozoan cnidarians.

### Mt-nuclear discordance

Advances in genomic approaches have also facilitated comparisons of the phylogenetic histories of nuclear and mt genomes. This has allowed for exploration of the patterns and underlying causes of mt-nuclear discordance. In both Hexacorallia and Octocorallia, we found a high degree of mt-nuclear discordance at every level (i.e., order to species) even when comparing the mt phylogeny to the most similar nuclear phylogeny. At deep nodes in the phylogenies, the most apparent differences in the hexacoral phylogenies included the positions of the anemone groups Actiniaria, Zoantharia, and *R. daphneae.* Within octocorals, the most apparent differences at deep nodes were relationships among clades within the order Scleralcyonacea and among the early-diverging lineages within Malacalcyonacea. Discordance at deep nodes complicates interpretations of ancestral state reconstructions through deep time. In addition, this level of discordance causes concern for using just one source of sequence data (i.e. nuclear or whole mt genomes) for phylogenetic reconstruction, but also highlights how different datasets used in compliment present a unique opportunity to better understand the cause of the discordance from an evolutionary perspective.

Substitution saturation of mt genomes has been suggested to be the cause of mt-nuclear discordance in anthozoans^21,41^. Using entropy tests on our extensive dataset of ~ 100 genomes in each class, we did not find evidence for substitution saturation. The entropy-based t statistic tests saturation on variable sites only, is suitable for assessing misleading tree topologies, and it has several advantages, including: (1) it is robust across a range of confounding factors, including rate variation across sites; and (2) the negative influence of slowly-evolving sites is removed in the measurement of overall base composition^50^. Thus, our results might differ from prior studies that used other methods, particularly if slowly-evolving sites were not taken into account. Alternatively, the different results could be driven by the number (2–3X less) and choice of taxa used in prior phylogenetic studies. In contrast to mt genomes, we found that ~ 10 to 50% of UCE and exon nuclear loci were saturated, depending on dataset. A recent study examining substitution saturation of UCE and exon loci across a range of taxa (e.g., hymenopterans, fishes, and crustaceans), also found similar numbers of saturated loci and noted that this could be driven by the highly variable flanking regions of UCEs^50^. We removed UCE and exon loci with substitution saturation from the dataset prior to phylogenetic analysis, yet even so, the nuclear and mt topologies were quite incongruent. Therefore, substitution saturation is not the primary cause of the observed discordance among nuclear and mt phylogenies.

Introgression is another biological process that can result in discordance among nuclear and mt phylogenies. Within Anthozoa, introgressive hybridization has been suggested to be an important mechanism in generating species diversity^72–78^. Because mt genomes are maternally inherited and non-recombining, species or groups of species that have undergone past hybridization might be expected to have mt genomes that are more similar than their nuclear genomes (e.g.^79,80^]). Using D-statistics and ABBA-BABA tests, Quattrini et al.^76^ determined that hybridization is an important mechanism in shaping diversity within the octocoral genus *Sclerophytum (*= *Sinularia*)*.* Similarly, hybridization has been noted within multiple species in the scleractinian genus *Porites*^77,78^. Indeed, we found strong incongruence between mt and nuclear phylogenies within both genera*.* Although incomplete lineage sorting is likely driving some incongruence at shallow nodes, our results and past data also suggest that introgression explains some of the incongruence, at least at the tips of the trees. Mitochondrial introgression is more likely and happens at a faster rate than nuclear introgression, cautioning the use of mt gene trees as accurate depictions of species trees^80^. Future studies should consider explicitly testing for mt introgression in pairs or groups of taxa using, for example, ABBA-BABA tests and isolation with migration models (e.g.^81^). Whether or not introgressive hybridization is the cause of incongruent relationships at nodes deeper in a phylogeny is more difficult to discern. However, ancient introgression of ghost lineages (e.g., extinct, unknown or unsampled lineages that remain in extant species likely due to ancient hybridization^82^) could play a role in generating incongruence and could be explored in future studies.

Unique properties of anthozoan mt genomes could also be partly responsible for the mt-nuclear discordance seen here. Anthozoan mt genomes evolve slowly^24,80^. This slow substitution rate can be seen clearly across both hexacoral and octocoral mt phylogenies as short branch lengths, particularly at the tips. Shearer et al.^80^ hypothesized that background selection is influencing the slow substitution rates within mt genomes of anthozoans. Due to non-recombining mt loci, selection reduces variation not only at sites under selection, but at those that are linked as well^83^. Indeed, we found that mt genomes are under strong purifying selection in both Hexacorallia and Octocorallia, with omega values close to zero and high kappa values suggesting transition bias in both classes. Another recent study found that some genes are under relaxed purifying selection in deep-sea taxa, with some sites in particular genes under positive selection^84^. We were not able to test for selection on nuclear loci, as none have been annotated to date, thus they are not in correct reading frames. However, because of the large number of loci used, we would not anticipate that all or even most nuclear loci would evolve under the same type of selection.

We also found variation in substitution rates and GC content across mt genome phylogenies and between mt and nuclear phylogenies. Although some relationships among species that occurred on long branches in the mt genome tree (e.g., *Tenerodus fallax* and *Leptophyton benayahui)* were also recovered in the nuclear tree, others were not (e.g., *Cornularia pabloi).* In a family-level revision of Octocorallia, McFadden et al.^40^ also noted that some species relationships in the gene tree for *mtMutS* were artifacts of long branch attraction, and that this rate variation among lineages influenced phylogenetic signal in mt data. Furthermore, we also showed pronounced differences in GC content between nuclear and mt data and even between different taxonomic orders. However, there are no obvious indications that GC content is driving discordance between nuclear and mt phylogenies.

### Summary

Our results have demonstrated pervasive mt-nuclear discordance in Anthozoa. Overall, non-recombining mt genomes that do not evolve neutrally, exhibit substantial rate variation, and are likely to rapidly introgress are most likely influencing our ability to reconstruct accurate species relationships using mt genome data alone. Other studies have cautioned against the use of mtDNA for resolving phylogenetic relationships in anthozoans ^21,41^ and even more broadly in metazoans^1^, but unequal taxon sampling and non-matching tips have always been potential confounding issues in mt-nuclear comparisons. We included the same tips in the mt and nuclear phylogenies and sampled widely across all orders. Nonetheless, it is still possible that inadequate taxon sampling could influence the patterns of mt-nuclear discordance we observed, and including more taxa in particular regions of the trees would stabilize some relationships. Even so, mt-nuclear discordance in hexacorals and octocorals is not an artifact of biased and incomparable taxon sampling, but is instead a signal of evolutionary processes that have shaped the genetic diversity of Anthozoa.

## Supplementary Information

Supplementary Figure 1.

Supplementary Figure 2.

Supplementary Table 1.

Supplementary Table 2.

Supplementary Table 3.

## Data Availability

Alignments, Tree files and code can be found on figshare 10.25573/data.21525147. Mitochondrial genes have been uploaded to GenBank and respective numbers can be found in supplemental tables.

## References

[1] Ballard JWO, Whitlock MC (2004). The incomplete natural history of mitochondria. Mol. Ecol..

[2] Janiak MC (2022). 205 newly assembled mitogenomes provide mixed evidence for rivers as drivers of speciation for Amazonian primates. Mol. Ecol..

[3] Allio R (2020). MitoFinder: Efficient automated large-scale extraction of mitogenomic data in target enrichment phylogenomics. Mol. Ecol. Res..

[4] Xiao M (2019). Mitogenomics suggests a sister relationship of *Relicanthus*
*daphneae* (Cnidaria: Anthozoa: Hexacorallia: *incerti*
*ordinis*) with Actiniaria. Sci. Rep..

[5] Brown WM, George M, Wilson AC (1979). Rapid evolution of animal mitochondrial DNA. Proc. Natl. Acad. Sci. U.S.A..

[6] Platt RN (2018). Conflicting evolutionary histories of the mitochondrial and nuclear genomes in new world *Myotis* bats. Syst. Biol..

[7] Tamashiro RA (2019). What are the roles of taxon sampling and model fit in tests of cyto-nuclear discordance using avian mitogenomic data?. Mol. Phylogenet. Evol..

[8] Kimball RT, Guido M, Hosner PA, Braun EL (2021). When good mitochondria go bad: Cyto-nuclear discordance in landfowl (Aves: Galliformes). Gene.

[9] Lavrov DV, Pett W (2016). Animal mitochondrial DNA as we do not know it: Mt-genome organization and evolution in nonbilaterian lineages. Genome. Biol. Evol..

[10] Beagley CT, Okada NA, Wolstenholme DR (1996). Two mitochondrial group I introns in a metazoan, the sea anemone *Metridium*
*senile*: One intron contains genes for subunits 1 and 3 of NADH dehydrogenase. PNAS.

[11] van Oppen MJ, Catmull J, McDonald BJ, Hislop NR, Hagerman PJ, Miller DJ (2002). The mitochondrial genome of *Acropora*
*tenuis* (Cnidaria; Scleractinia) contains a large group I intron and a candidate control region. J. Mol. Evol..

[12] Medina M, Collins AG, Takaoka TL, Kuehl JV, Boore JL (2006). Naked corals: Skeleton loss in Scleractinia. PNAS.

[13] Fukami H, Chen CA, Chiou CY, Knowlton N (2007). Novel group I introns encoding a putative homing endonuclease in the mitochondrial cox1 gene of Scleractinian corals. J. Mol. Evol..

[14] Brugler MR, France SC (2007). The complete mitochondrial genome of the black coral *Chrysopathes*
*formosa* (Cnidaria: Anthozoa: Antipatharia) supports classification of antipatharians within the subclass Hexacorallia. Mol. Phylogenet. Evol..

[15] Stampar SN (2019). Linear mitochondrial genome in Anthozoa (Cnidaria): A case study in Ceriantharia. Sci. Rep..

[16] Muthye V, Mackereth CD, Stewart JB, Lavrov DV (2022). Large dataset of octocoral mitochondrial genomes provides new insights into *mt-mutS* evolution and function. DNA Repair.

[17] Bilewitch JP, Degnan SM (2011). A unique horizontal gene transfer event has provided the octocoral mitochondrial genome with an active mismatch repair gene that has potential for an unusual self-contained function. BMC Evol. Biol..

[18] Hogan RI, Hopkins K, Wheeler AJ, Allcock AL, Yesson C (2019). Novel diversity in mitochondrial genomes of deep-sea Pennatulacea (Cnidaria: Anthozoa: Octocorallia). Mitochondrial DNA Part A.

[19] Uda K (2011). Complete mitochondrial genomes of two Japanese precious corals, *Paracorallium*
*japonicum* and *Corallium*
*konojoi* (Cnidaria, Octocorallia, Coralliidae): Notable differences in gene arrangement. Gene.

[20] Brockman SA, McFadden CS (2012). The mitochondrial genome of *Paraminabea aldersladei* (Cnidaria: Anthozoa: Octocorallia) supports intramolecular recombination as the primary mechanism of gene rearrangement in octocoral mitochondrial genomes. Genome Biol. Evol..

[21] Figueroa DF, Baco AR (2015). Octocoral mitochondrial genomes provide insights into the phylogenetic history of gene order rearrangements, order reversals, and cnidarian phylogenetics. Genome Biol. Evol..

[22] Brown WM, Prager EM, Wang A, Wilson AC (1982). Mitochondrial DNA sequences of primates: Tempo and mode of evolution. J. Mol. Evol..

[23] Vawter L, Brown WM (1986). Nuclear and mitochondrial DNA comparisons reveal extreme rate variation in the molecular clock. Science.

[24] Hellberg ME (2006). No variation and low synonymous substitution rates in coral mtDNA despite high nuclear variation. BMC Evol. Biol..

[25] Shearer TL, Coffroth MA (2008). DNA Barcoding: Barcoding corals: limited by interspecific divergence, not intraspecific variation. Mol. Ecol Res..

[26] Huang D, Meier R, Todd PA, Chou LM (2008). Slow mitochondrial COI sequence evolution at the base of the metazoan tree and its implications for DNA barcoding. J. Mol. Evol..

[27] Quattrini AM (2020). Palaeoclimate ocean conditions shaped the evolution of corals and their skeletons through deep time. Nat. Ecol. Evol..

[28] McFadden CS (2021). Phylogenomics, origin, and diversification of anthozoans (Phylum Cnidaria). Syst. Biol..

[29] Park E (2012). Estimation of divergence times in cnidarian evolution based on mitochondrial protein-coding genes and the fossil record. Mol. Phylogenet. Evol..

[30] Kayal E, Roure B, Philippe H, Collins AG, Lavrov DV (2013). Cnidarian phylogenetic relationships as revealed by mitogenomics. BMC Evol. Biol..

[31] Daly M (2007). The phylum Cnidaria: A review of phylogenetic patterns and diversity 300 years after Linnaeus. Zootaxa.

[32] Zapata F (2015). Phylogenomic analyses support traditional relationships within Cnidaria. PLoS ONE.

[33] Kayal E (2018). Phylogenomics provides a robust topology of the major cnidarian lineages and insights on the origins of key organismal traits. BMC Evol. Biol..

[34] Osigus H-J, Eitel M, Bernt M, Donath A, Schierwater B (2013). Mitogenomics at the base of metazoa. Mol. Phylogenet. Evol..

[35] Stampar SN, Maronna MM, Kitahara MV, Reimer JD, Morandini AC (2014). Fast-evolving mitochondrial DNA in Ceriantharia: A reflection of Hexacorallia paraphyly?. PLoS ONE.

[36] Kitahara MV (2014). The “Naked Coral” hypothesis revisited: Evidence for and against Scleractinian monophyly. PLoS ONE.

[37] Seiblitz IGL (2020). The earliest diverging extant scleractinian corals recovered by mitochondrial genomes. Sci. Rep..

[38] Stolarski J (2021). A modern scleractinian coral with a two-component calcite–aragonite skeleton. PNAS.

[39] McFadden CS, France SC, Sánchez JA, Alderslade P (2006). A molecular phylogenetic analysis of the Octocorallia (Cnidaria: Anthozoa) based on mitochondrial protein-coding sequences. Mol. Phylogenet. Evol..

[40] McFadden CS, van Ofwegen LP, Quattrini AM (2022). Revisionary systematics of Octocorallia (Cnidaria: Anthozoa) guided by phylogenomics. Bull. Syst. Biol..

[41] Pratlong M, Rancurel C, Pontarotti P, Aurelle D (2016). Monophyly of Anthozoa (Cnidaria): Why do nuclear and mitochondrial phylogenies disagree?. Zool. Scr..

[42] Quattrini AM (2018). Universal target-enrichment baits for anthozoan (Cnidaria) phylogenomics: New approaches to long-standing problems. Mol. Ecol. Resour..

[43] Faircloth, B. C. Illumiprocessor: a trimmomatic wrapper for parallel adapter and quality trimming. 10.6079/J9ILL (2013).

[44] Bolger AM, Lohse M, Usadel B (2014). Trimmomatic: A flexible trimmer for Illumina sequence data. Bioinformatics.

[45] Bankevich A (2012). SPAdes: A new genome assembly algorithm and its applications to single-cell sequencing. J. Comput. Biol..

[46] Haas BJ (2013). De novo transcript sequence reconstruction from RNA-seq using the Trinity platform for reference generation and analysis. Nat. Protoc..

[47] Katoh K, Standley DM (2013). MAFFT multiple sequence alignment software version 7: Improvements in performance and usability. Mol. Biol. Evol..

[48] Dierckxsens N, Mardulyn P, Smits G (2016). NOVOPlasty: *De novo* assembly of organelle genomes from whole genome data. Nucleic Acids Res..

[49] Donath A (2019). Improved annotation of protein-coding genes boundaries in metazoan mitochondrial genomes. Nucleic Acids Res..

[50] Duchêne DA, Mather N, van der Wal C, Ho SYW (2021). Excluding loci with substitution saturation improves inferences from phylogenomic data. Syst. Biol..

[51] Duchêne DA, Duchêne S, Ho SYW (2018). PhyloMAd: Efficient assessment of phylogenomic model adequacy. Bioinformatics.

[52] Yang Z (2007). PAML 4: Phylogenetic analysis by maximum likelihood. Mol. Biol. Evol..

[53] Nguyen L-T, Schmidt HA, von Haeseler A, Minh BQ (2015). IQ-TREE: A fast and effective stochastic algorithm for estimating maximum-likelihood phylogenies. Mol. Biol. Evol..

[54] Kalyaanamoorthy S, Minh BQ, Wong TKF, von Haeseler A, Jermiin LS (2017). ModelFinder: Fast model selection for accurate phylogenetic estimates. Nat. Methods.

[55] Hoang DT, Chernomor O, von Haeseler A, Minh BQ, Vinh LS (2018). UFBoot2: Improving the ultrafast bootstrap approximation. Mol. Biol. Evol..

[56] Anisimova M, Gil M, Dufayard J-F, Dessimoz C, Gascuel O (2011). Survey of branch support methods demonstrates accuracy, power, and robustness of fast likelihood-based approximation schemes. Syst. Biol..

[57] Minh BQ, Hahn MW, Lanfear R (2020). New methods to calculate concordance factors for phylogenomic datasets. Mol. Biol. Evol..

[58] Zhang C, Rabiee M, Sayyari E, Mirarab S (2018). ASTRAL-III: polynomial time species tree reconstruction from partially resolved gene trees. BMC Bioinform..

[59] Mai U, Mirarab S (2018). TreeShrink: fast and accurate detection of outlier long branches in collections of phylogenetic trees. BMC Genom..

[60] Revell LJ (2012). phytools: an R package for phylogenetic comparative biology (and other things). Methods. Ecol. Evol..

[61] Robinson DF, Foulds LR (1981). Comparison of phylogenetic trees. Math Biosci..

[62] Smith MR (2022). Robust analysis of phylogenetic tree space. Syst. Biol..

[63] Shen W, Le S, Li Y, Hu F (2016). SeqKit: A cross-platform and ultrafast toolkit for FASTA/Q file manipulation. PLoS ONE.

[64] Wickham H (2011). ggplot2. Wiley Interdiscip. Rev..

[65] Poliseno A (2020). Evolutionary implications of analyses of complete mitochondrial genomes across order Zoantharia (Cnidaria: Hexacorallia). J. Zool. Syst. Evol. Res..

[66] Emblem Å (2014). Sea anemones possess dynamic mitogenome structures. Mol. Phylogenet. Evol..

[67] Celis JS (2017). Evolutionary and biogeographical implications of degraded LAGLIDADG endonuclease functionality and group I intron occurrence in stony corals (Scleractinia) and mushroom corals (Corallimorpharia). PLoS ONE.

[68] Foox J, Brugler M, Siddall ME, Rodríguez E (2016). Multiplexed pyrosequencing of nine sea anemone (Cnidaria: Anthozoa: Hexacorallia: Actiniaria) mitochondrial genomes. Mitochondrial DNA A.

[69] Seiblitz IG (2022). Caryophylliids (Anthozoa, Scleractinia) and mitochondrial gene order: Insights from mitochondrial and nuclear phylogenomics. Mol. Phyl. Evol..

[70] Brugler MR, France SC (2008). The mitochondrial genome of a deep-sea bamboo coral (Cnidaria, Anthozoa, Octocorallia, Isididae): genome structure and putative origins of replication are not conserved among octocorals. J. Mol. Evol..

[71] Johansen SD, Emblem Å (2020). Mitochondrial Group I introns in hexacorals are regulatory genetic elements. Advances in the Studies of the Benthic Zone.

[72] van Oppen MV, Willis BL, Vugt HV, Miller DJ (2000). Examination of species boundaries in the *Acropora*
*cervicornis* group (Scleractinia, Cnidaria) using nuclear DNA sequence analyses. Mol. Ecol..

[73] Vollmer SV, Palumbi SR (2002). Hybridization and the evolution of reef coral diversity. Science.

[74] Reimer JD, Takishita K, Ono S, Tsukahara J, Maruyama T (2007). Molecular evidence suggesting interspecific hybridization in *Zoanthus* spp. (Anthozoa: Hexacorallia). Zool. Sci..

[75] Combosch DJ, Vollmer SV (2015). Trans-Pacific RAD-Seq population genomics confirms introgressive hybridization in Eastern Pacific *Pocillopora* corals. Mol. Phylogenet. Evol..

[76] Quattrini AM (2019). A next generation approach to species delimitation reveals the role of hybridization in a cryptic species complex of corals. BMC Evol. Biol..

[77] Hellberg ME, Prada C, Tan MH, Forsman ZH, Baums IB (2016). Getting a grip at the edge: Recolonization and introgression in eastern Pacific *Porites* corals. J. Biogeogr..

[78] Forsman ZH (2017). Coral hybridization or phenotypic variation? Genomic data reveal gene flow between *Porites*
*lobata* and *P.*
*compressa*. Mol. Phylogenet. Evol..

[79] Chan KM, Levin SA (2005). Leaky prezygotic isolation and porous genomes: rapid introgression of maternally inherited DNA. Evol..

[80] Shearer TL, Van Oppen MJH, Romano SL, Wörheide G (2002). Slow mitochondrial DNA sequence evolution in the Anthozoa (Cnidaria). Mol. Ecol..

[81] Hey J (2010). Isolation with migration models for more than two populations. Mol. Biol. Evol..

[82] Hibbins MS, Hahn MW (2022). Phylogenomic approaches to detecting and characterizing introgression. Genetics.

[83] Schrider DR (2020). Background selection does not mimic the patterns of genetic diversity produced by selective sweeps. Genetics.

[84] Ramos NI, DeLeo DM, McFadden CS, Quattrini AM (2023). Selection in coral mitogenomes, with insights into adaptations in the deep sea. Sci. Rep..

