# Supplementary material for: Mito-nuclear discordance within Anthozoa, with notes on unique properties of their mitochondrial genomes
Source: Sci Rep. 2023 May 8;13:7443. doi: 10.1038/s41598-023-34059-1 (PMC10167242; doi:10.1038/s41598-023-34059-1)
Supplement: Supplementary file 1 — Supplementary Figure 1. [file 41598_2023_34059_MOESM1_ESM.pdf]

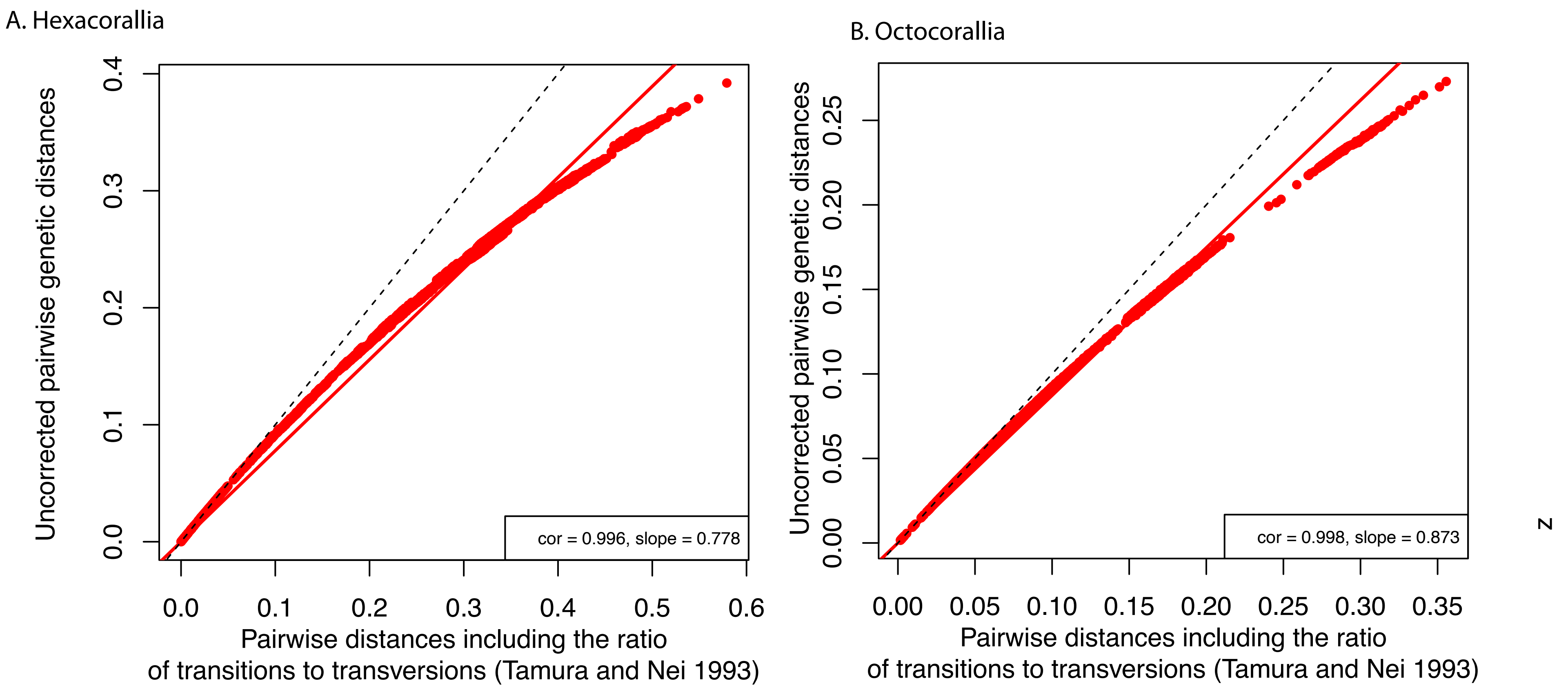

Suppl. Figure 1. Substitution saturation plots for A) Hexacorallia and B) Octocoralla.
