# Supplementary material for: Mito-nuclear discordance within Anthozoa, with notes on unique properties of their mitochondrial genomes
Source: Sci Rep. 2023 May 8;13:7443. doi: 10.1038/s41598-023-34059-1 (PMC10167242; doi:10.1038/s41598-023-34059-1)
Supplement: Supplementary file 2 — Supplementary Figure 2. [file 41598_2023_34059_MOESM2_ESM.pdf]

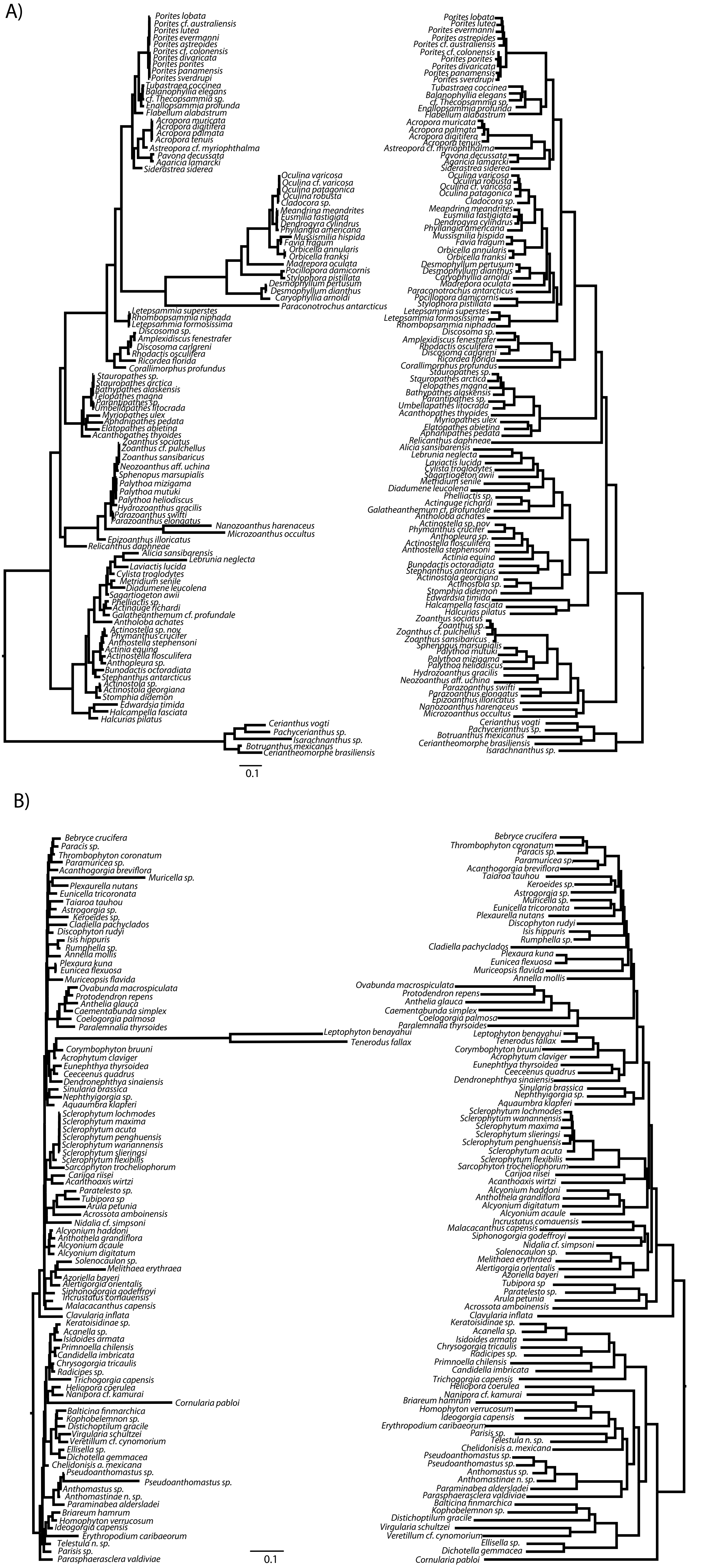

Suppl. Figure 2. Mt genome (left) and nuclear (right) phylogenies for A) Hexacorallia and B) Octocorallia. Topologies match figures 2 and 3, but branch lengths are shown here.
